# Risk of cardiovascular disease among different fluoropyrimidine-based chemotherapy regimens as adjuvant treatment for resected colorectal cancer

**DOI:** 10.3389/fcvm.2022.880956

**Published:** 2022-08-03

**Authors:** Wen-Kuan Huang, Wei-Pang Ho, Hung-Chih Hsu, Shu-Hao Chang, Dong-Yi Chen, Wen-Chi Chou, Pei-Hung Chang, Jen-Shi Chen, Tsai-Sheng Yang, Lai-Chu See

**Affiliations:** ^1^Division of Hematology-Oncology, Department of Internal Medicine, Chang Gung Memorial Hospital at Linkou, Taoyuan, Taiwan; ^2^College of Medicine, Chang Gung University, Taoyuan, Taiwan; ^3^Department of Public Health, College of Medicine, Chang Gung University, Taoyuan, Taiwan; ^4^Division of Cardiology, Department of Internal Medicine, Chang Gung Memorial Hospital at Linkou, Taoyuan, Taiwan; ^5^Division of Hematology-Oncology, Department of Internal Medicine, Chang Gung Memorial Hospital at Keelung, Keelung, Taiwan; ^6^Division of Rheumatology, Allergy and Immunology, Department of Internal Medicine, Chang Gung Memorial Hospital at Linkou, Taoyuan, Taiwan; ^7^Biostatistics Core Laboratory, Molecular Medicine Research Center, Chang Gung University, Taoyuan, Taiwan

**Keywords:** cardiovascular disease, fluoropyrimidine, colorectal cancer, mortality, adjuvant chemotherapy

## Abstract

**Background:**

Patients with colorectal cancer (CRC) are more likely to develop cardiovascular disease (CVD) than those without cancer. Little is known regarding their CV risk after operative chemotherapy. We aimed to compare the risk of CV disease among different fluoropyrimidine derivatives.

**Methods:**

We assembled a nationwide cohort of patients with newly diagnosed CRC between 2004 and 2015 who received fluoropyrimidine-based adjuvant chemotherapy for resected CRC by linking the Taiwan Cancer Registry (TCR), National Health Insurance Research Database (NHIRD), and Taiwan Death Registry (TDR). All eligible patients were followed from CRC diagnosis (index date) until a CV event, death, loss to follow-up, or December 31st 2018, whichever came first. CV outcomes included acute myocardial infarction (AMI), life-threatening arrhythmia (LTA), congestive heart failure (CHF), and ischemic stroke (IS). We used stabilized inverse probability of treatment weighting using propensity score (SIPTW) to balance all covariates among the three chemotherapy groups: tegafur-uracil (UFT), non-UFT, and mixed. In addition, survival analysis was conducted to examine the association between study outcomes and chemotherapy groups.

**Results:**

From 2004 to 2015, 10,615 (32.8%) patients received UFT alone, 14,511 (44.8%) patients received non-UFT, and 7,224 (22.3%) patients received mixed chemotherapy. After SIPTW, the UFT group had significantly lower all-cause mortality and cancer-related death rates than the other two chemotherapy groups. However, the UFT group had significantly higher rates of cancer death, ischemic stroke, and heart failure than those of the other two chemotherapy groups. The UFT group also had a significantly higher AMI rate than the mixed group. There was no significant difference in LTA among the three groups. Similar findings were observed in the subgroup analysis (stage II and age <70 years, stage II and age ≥70 years, stage III and age <70 years, stage III and age ≥70 years) as the overall population was observed.

**Conclusion:**

Higher heart failure and ischemic stroke rates were found in the UFT group than in the other two chemotherapy groups, especially those with stage III CRC and ≥70 years of age. Careful monitoring of this subset of patients when prescribing UFT is warranted.

## Introduction

Over the past two decades, early-stage cancer detection and treatment improvements have significantly improved the prognosis of several major cancers, such as colorectal cancer (CRC), prostate cancer, and breast cancer (1). It has been postulated that the population of cancer survivors in the USA will increase to eighteen million by 2022 (2). Within the population of cancer survivors, the awareness of health problems that can occur after cancer survival is increasing. Among these, treatment-related cardiovascular diseases are a major concern (3). The US Surveillance, Epidemiology, and End Results (SEER) study showed that cardiovascular (CV) death was the most common cause of non-cancer deaths among cancer patients in 1973–2012 (4). One cohort study, with 36,232 2-year survivors from 2000 to 2007 who were followed up until 2012, found that cancer survivors had significantly more CV adverse events than non-cancer controls (5).

Patients with colorectal cancer are more likely to develop cardiovascular disease (CVD) than those without cancer (6). Kenzik et al. conducted a US population-based study to determine the long-term risk of cardiovascular disease (including stroke and myocardial infarction) and congestive heart failure (CHF) in stage I–III CRC survivors aged 65 years. The 10-year cumulative incidence of new-onset cardiovascular disease and CHF was 57.4 and 54.5% for patients with stage I–III CRC compared with 22 and 18% for the matched cohort without cancer, respectively (*p* < 0.001) (7). Correspondingly, a Korean cohort study reported 141 (4.9%) patients who developed new-onset CVD among postoperative CRC patients (8). These studies raised the increasing concern of CV-related adverse events among CRC survivors.

**Figure 1:**
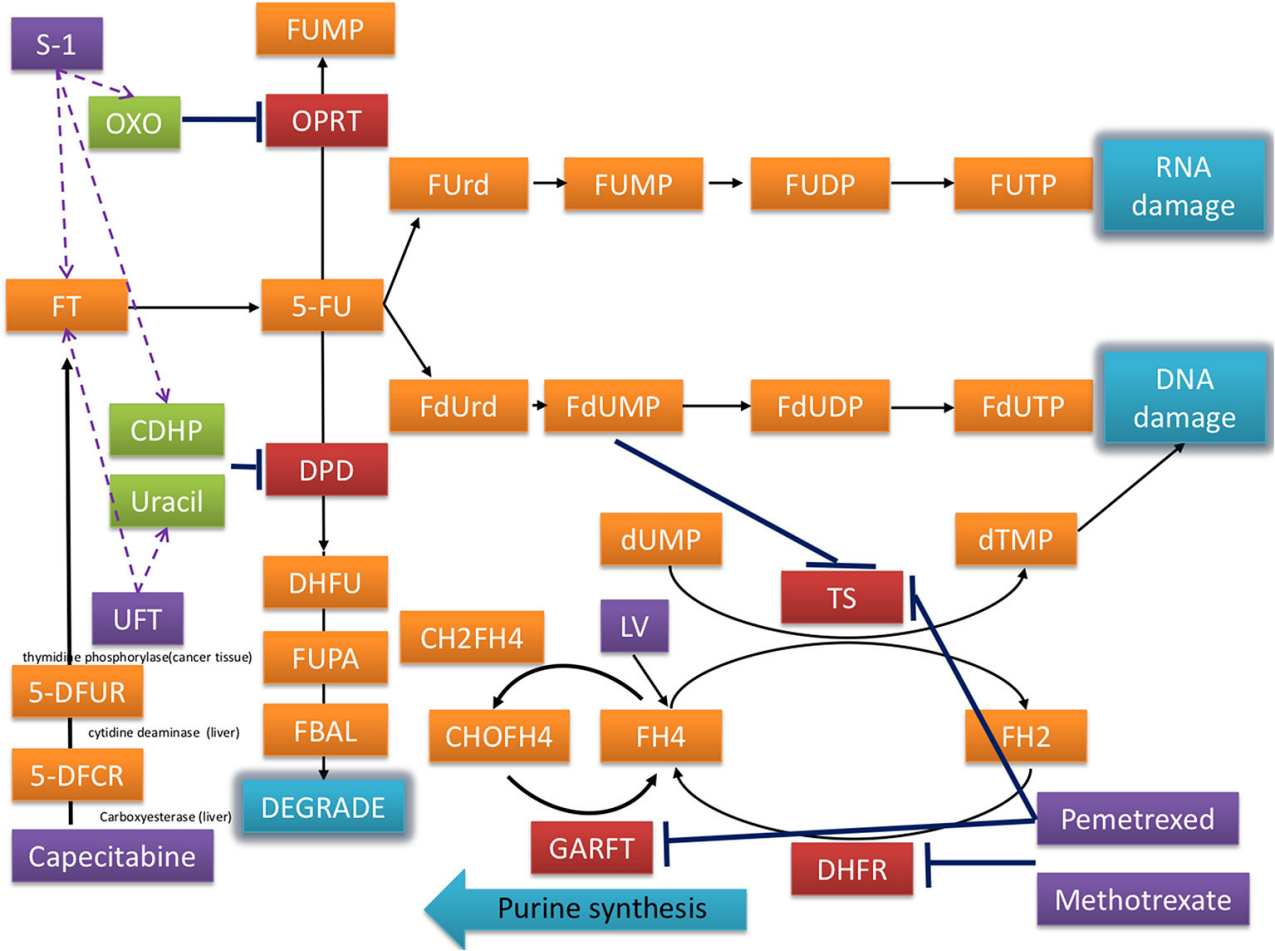
Mechanisms of fluorouracil-related medications including UFT, capecitabine, S-1, and others in the treatment of CRC.

Postoperative chemotherapy is associated with an increased risk of CVD (7, 8). Fluoropyrimidine is the backbone of chemotherapy in the adjuvant setting in patients with CRC. In addition to intravenous 5- fluorouracil (FU), oral fluoropyrimidine including UFT, TS-1 and capecitabine were commonly used in Asian countries (9, 10). While these oral prodrugs were finally metabolized to 5-FU, their adverse events were somewhat different, which may be due to the components of prodrugs (Figure 1). For example, gimeracil (the compoenent of TS-1) and uracil (the component of UFT) inhibit dihydropyrimidine dehydrogenase, which degrades 5-FU, leading to enhance cytotoxic effects.

Notably, exposure to fluoropyrimidine increases the risk of CV in patients with cancer (11). However, whether fluoropyrimidine derivatives including intravenous 5-FU, capecitabine, and tegafur-uracil (UFT) have a different effect on cardiotoxicity remains unclear. UFT is an oral agent in which uracil competes with dihydropyrimidine dehydrogenase, which reduces the catabolism of 5-FU and its cardiotoxic metabolites. No study has compared UFT with other fluoropyrimidine derivatives concerning their subsequent CVD risk. The Taiwan Cancer Registry (TCR), National Death Registry (NDR), and National Health Insurance Research Database (NHIRD) provide comprehensive and accurate information on the diagnosis, staging, treatment, and survival of cancer patients in Taiwan. Here, we linked the above databases to evaluate the cardiotoxicity of different 5-FU derivatives in the adjuvant setting for patients with CRC.

## Methods

### Data sources

The data sources for this study included the TCR, NHIRD, and TDR. These three databases are linked with encrypted personal identification numbers and are available at the Health and Welfare Data Center (HWDC). In addition, we obtained approval from the IRB of the Chang Gung Medical Foundation, Taiwan (201901844B0). The need for informed consent was waived because the personal ID had already been encrypted.

### Study design

**Figure 2:**
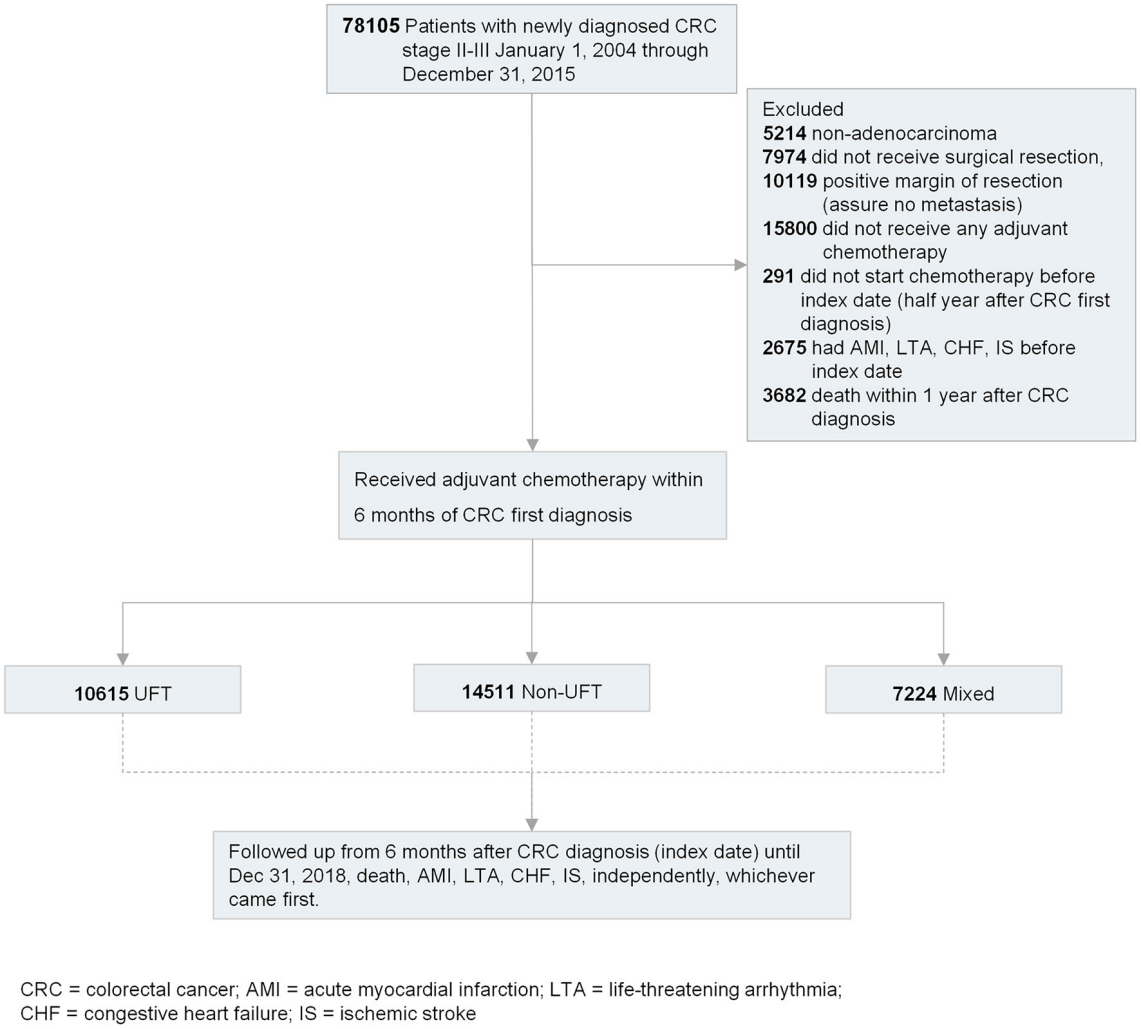
Flowchart of enrolling study participants and follow-up for study outcomes.

We established a nationwide cohort of patients with newly diagnosed stage II–III CRC in 2004–2015 and received FU-based adjuvant chemotherapy for resection. All eligible patients were followed up from 6 months after the first diagnosis of CRC (index date) until the occurrence of cardiotoxicity (AMI, LTA, CHF, IS, independently), death, and loss to follow-up on December 31st 2018, whichever came first. The index date was set 6 months after CRC first diagnosis because (1) the majority of patients with stage II–III CRC had their tumor removed surgically and started FU-based adjuvant chemotherapy within 6 months after the initial diagnosis of CRC, and (2) all eligible patients were followed equally with the same initial time point to reduce immortal time bias (Figure 2) (12).

The cohort was divided into three adjuvant chemotherapy groups: UFT, non-UFT, and mixed. Patients were excluded if they (1) had non-adenocarcinomatous CRC; (2) did not receive surgical resection; (3) had a positive resection margin; (4) did not receive any adjuvant chemotherapy; (5) did not start chemotherapy before the index date (6 months after initial CRC diagnosis); (6) had AMI, LTA, CHF, or IS before the index date; (7) missing sex and birth year data; and (8) implausible data, such as death before CRC diagnosis or inconsistent initial date of adjuvant chemotherapy in TCR, NHIRD, and TDR (Figure 2).

### Outcomes

The study outcomes were as follows: (1) mortality, all-cause mortality, cancer mortality, CV mortality, and non-CV mortality; and (2) CV events including acute myocardial infarction (AMI), life-threatening arrhythmia (LTA), congestive heart failure (CHF), and ischemic stroke (IS). Furthermore, to reduce misclassification, all CV outcomes had to be the principal diagnosis of hospitalization admission or the first diagnosis through the emergency department based on ICD-9 (until 2015) or ICD-10 (since 2016) (Supplementary Table 1).

### Covariates

For demographic characteristics, we obtained age, sex, income level, and enrollment category from the NHIRD. In addition, we obtained the calendar year of CRC diagnosis, primary site, stage, tumor grade, and tumor treatment modality from the TCR for cancer-related characteristics. For comorbidities, we obtained data on hypertension, dyslipidemia, diabetes, coronary artery disease, chronic obstructive pulmonary disease (COPD), peripheral arterial disease, chronic kidney disease, atrial fibrillation, and moderate or severe liver disease from the NHIRD. All comorbidities had to occur 1 year before the first diagnosis of CRC. Regarding CV-related medication, we were confined to metformin, aspirin, and statins, which were prescribed 1 year before the first diagnosis of CRC from the NHIRD (Supplementary Table 1).

### Statistical analysis

We first balanced all covariates among the three chemotherapy groups by stabilizing the inverse probability of treatment weighting using the propensity score (SIPTW) (13). The advantage of using SIPTW is obtaining an appropriate estimation of the variance of the main effect (average treatment effect for the population, ATE) and maintaining an adequate type I error by preserving the sample size of the original data. In SIPTW, we used a generalized boosted model (GBM) (14) to compute the propensity score because GBM gives the best performance in various scenarios (additivity and linearity, mild non-additivity and non-linearity, and moderate non-additivity and non-linearity in different weight trimming percentiles) (15), and can be extended to more than two treatment groups (14). The covariates in Table 1 were included in the GBM. Next, the absolute standardized mean difference (ASMD) was used to assess the balance of covariates at baseline (index date) among the three chemotherapy groups. A maximum value of ASMD ≤ 0.1 indicated an insignificant difference in covariates among the three chemotherapy groups (16).

**Table 1:** Demographic, cancer, comorbidity, and medication characteristics among patients with stage II–III colorectal cancer before SIPTW.

	**Before SIPTW**			
	**UFT (*n* = 10,615)**	**Non-UFT (*n* = 14,511)**	**Mixed (*n* = 7,224)**	**ASMD**
**Age at diagnosis, years**				0.5249
Median (Q1–Q3)	67.72 (12.47)	61.24 (12.24)	62.12 (12.49)	
Mean (SD)	69 (18)	61 (17)	62.5 (18)	
Range	12–99	14–99	17–97	
<50	905 (8.53%)	2,376 (16.37%)	1,134 (15.7%)	0.5075
50–59	1,868 (17.6%)	3,989 (27.49%)	1,824 (25.25%)	
60–69	2,545 (23.98%)	4,182 (28.82%)	2,017 (27.92%)	
≥70	5,297 (49.90%)	3,964 (25.72%)	2,249 (31.13%)	
**Gender**				0.0127
Men	5,950 (56.05%)	8,225 (56.68%)	4,089 (56.6%)	
Women	4,665 (43.95%)	6,286 (43.32%)	3,135 (43.4%)	
**Enrollee category**				0.1274
EC1	780 (7.35%)	1,228 (8.46%)	552 (7.64%)	
EC2	2,806 (26.43%)	4,477 (30.85%)	2,105 (29.14%)	
EC3	4,414 (41.58%)	5,686 (39.18%)	3,000 (41.53%)	
EC4	2,615 (24.63%)	3,120 (21.5%)	1,567 (21.69%)	
**Income level**				0.2410
Dependent (quartile1)	3,771 (35.53%)	4,507 (31.06%)	2,320 (32.12%)	
<15,000 (quartile2)	1,981 (18.66%)	2,415 (16.64%)	1,200 (16.61%)	
15,000–24,999 (quartile3)	3,449 (32.49%)	4,421 (30.47%)	2,327 (32.21%)	
≥25,000 (quartile4)	1,414 (13.32%)	3,168 (21.83%)	1,377 (19.06%)	
**Year of diagnosis**				0.0870
2004–2006	1,524 (14.36%)	2,472 (17.04%)	1,163 (16.1%)	
2007–2010	3,478 (32.76%)	4,811 (33.15%)	2,396 (33.17%)	
2011–2014	5,613 (52.88%)	7,228 (49.81%)	3,665 (50.73%)	
**Primary site**				0.1776
Colon	6,442 (60.69%)	9,229 (63.6%)	4,055 (56.13%)	
Rectosigmoid	798 (7.52%)	1,265 (8.72%)	619 (8.57%)	
Rectum	3,375 (31.79%)	4,017 (27.68%)	2,550 (35.3%)	
**Stage**				0.9657
II	6,980 (65.76%)	2,747 (18.93%)	1,694 (23.45%)	
III	3,606 (33.97%)	11,731 (80.84%)	5,518 (76.38%)	
Unknown	29 (0.27%)	33 (0.23%)	12 (0.17%)	
**Grade**				0.1589
Well or moderately differentiated	9,564 (90.1%)	12,497 (86.12%)	6,108 (84.55%)	
Poorly differentiated	687 (6.47%)	1,387 (9.56%)	701 (9.7%)	
Unknown	364 (3.43%)	627 (4.32%)	415 (5.74%)	
**Primary treatment**				0.3264
OP alone	0 (0%)	0 (0%)	0 (0%)	
OP-CCRT	516 (4.86%)	1,010 (6.96%)	576 (7.97%)	
OP-CT	9,230 (86.95%)	11,738 (80.89%)	5,450 (75.44%)	
Neo-CCRT	428 (4.03%)	851 (5.86%)	676 (9.36%)	
Neo-CT	48 (0.45%)	57 (0.39%)	57 (0.79%)	
Unknown + missing	393 (3.7%)	855 (5.89%)	465 (6.44%)	
**Comorbidity**				
Hypertension	6,033 (56.83%)	6,747 (46.5%)	3,518 (48.7%)	0.208
Dyslipidemia	3,181 (29.97%)	3,118 (21.49%)	1,688 (23.37%)	0.1949
Coronary artery disease	3,106 (29.26%)	3,571 (24.61%)	1,783 (24.68%)	0.1050
Diabetes mellitus	3,999 (37.67%)	4,904 (33.8%)	2,449 (33.9%)	0.0810
Chronic obstructive pulmonary disease	308 (2.9%)	224 (1.54%)	140 (1.94%)	0.0922
Peripheral arterial disease	810 (7.63%)	718 (4.95%)	397 (5.5%)	0.1107
Chronic kidney disease	2,379 (22.41%)	2,353 (16.22%)	1,246 (17.25%)	0.1575
Atrial fibrillation	529 (4.98%)	400 (2.76%)	193 (2.67%)	0.1207
Moderate or severe liver disease	28 (0.26%)	26 (0.18%)	15 (0.21%)	0.0180
**Postdiagnostic medication**				
Aspirin	2,107 (19.85%)	2,066 (14.24%)	1,135 (15.71%)	0.1497
Metformin	1,339 (12.61%)	1,562 (10.76%)	777 (10.76%)	0.0579
Statin	1,726 (16.26%)	1,996 (13.76%)	1,023 (14.16%)	0.0702

*ASMD, absolute standardized mean difference; EC1, civil servants: full-time or regularly paid personnel with a government or public affiliation; EC2, employees of privately owned institutions; EC3, self-employed individuals, other employees, and members of farmers' or fishermen's associations; EC4, veterans, members of low-income families, and substitute service draftees; CCRT, chemotherapy and radiotherapy; CT, chemotherapy; OP, operation; SIPTW, stabilized inverse probability of treatment weighting using propensity score; UFT, fluoropyrimidine*.

Next, we performed survival analysis [log-rank test in univariate analysis, Cox's proportional hazard model (17) and cause-specific hazard model (18) in the multivariate analysis] to examine the association between study outcomes and chemotherapy groups. We treated death as a competing risk event in the cause-specific hazard model. In either Cox's or the cause-specific hazard models, chemotherapy (CT) grouping was the only covariate because the three chemotherapy groups were balanced after SIPTW (19). We plotted log {-log[S(t)]} vs. log(t) to check the proportional hazards assumption in the Cox and cause-specific hazard models, where S(t) is the cumulative survival over time t. The lines of the three CT grouping within each plot of log {-log [S(t)]} vs. log(t) were parallel, indicating no violation of proportional hazards (20).

We also performed subgroup analysis to examine whether the differences in study outcomes between the three CT groups were maintained in specific subgroups: stage II and age <70 years, stage II and age ≥70 years, stage III and age <70 years, and stage III and age ≥70 years. For each subgroup analysis, we re-estimated the SIPTW to ensure a balance of covariates across groups.

## Results

From 2004 to 2015, 78,105 patients were newly diagnosed with stage II–III CRC. Based on the exclusion criteria, 32,350 patients were eligible for this study. Among these 32,350 patients, 10,615 (32.8%) received UFT alone, 14,511 (44.8%) received non-UFT, and 7,224 (22.3%) received mixed chemotherapy (Figure 2). Before SIPTW, the UFT group was older, had a lower income; had better tumor grade; had a higher proportion of receiving OP-CT; had a higher prevalence of hypertension, coronary artery disease, diabetes, peripheral arterial disease, and COPD; and a higher aspirin prescription rate than those of the other two CT groups (Table 1). After SIPTW, the three CT groups were well-balanced in demographic characteristics, cancer-related characteristics, comorbidities, and CV-related medication (Table 2).

**Table 2:** Demographic, cancer, comorbidity, and medication characteristics among patients with stage II–III colorectal cancer after SIPTW.

	**After sIPTW**			
	**UFT (*n* = 9138.89)**	**Non-UFT** **(*n* = 12773.7)**	**Mixed** **(*n* = 6367.9)**	***P*** **-value**
**Age at diagnosis, years**				0.0776
Median (Q1–Q3)	64.19 (12.07)	63.24 (12.34)	63.35 (12.43)	
Mean (SD)	65(19)	64(18)	64(18)	
Range	12–99	14–99	17–97	
<50	1,213.11 (12.19%)	1,955.88 (13.9%)	968.85 (13.76%)	0.0972
50–59	2,319.08 (23.3%)	3,378.27 (24.01%)	1,692.20 (24.03%)	
60–69	2,694.03 (27.07%)	3,892.11 (27.66%)	1,929.81 (27.41%)	
≥70	2,912.67 (37.44%)	3,548.44 (34.43%)	1,777.04 (34.80%)	
**Gender**				0.0084
Men	5,613.07 (56.4%)	7,973.92 (56.67%)	4,000.61 (56.82%)	
Women	4,339.14 (43.6%)	6,097.22 (43.33%)	3,040.73 (43.18%)	
**Enrollee category**				0.0282
EC1	760.65 (7.64%)	1,091.26 (7.76%)	539.80 (7.67%)	
EC2	2,786.84 (28%)	4,083.52 (29.02%)	2,045.62 (29.05%)	
EC3	4,111.67 (41.31%)	5,758.37 (40.92%)	2,867.40 (40.72%)	
EC4	2,293.05 (23.04%)	3,137.99 (22.3%)	1,588.51 (22.56%)	
**Income level**				0.0558
Dependent (quartile 1)	3,316.54 (33.32%)	4,578.32 (32.54%)	2,320.54 (32.96%)	
<15,000 (quartile 2)	1,752.40 (17.61%)	2,419.03 (17.19%)	1,212.52 (17.22%)	
15,000–24,999 (quartile 3)	3,139.98 (31.55%)	4,454.99 (31.66%)	2,201.41 (31.26%)	
≥25,000 (quartile 4)	1,743.29 (17.52%)	2,618.80 (18.61%)	1,306.87 (18.56%)	
**Year of diagnosis**				0.0445
2004–2006	1,634.14 (16.42%)	2,242.34 (15.94%)	1,126.93 (16%)	
2007–2010	3,440.38 (34.57%)	4,661.65 (33.13%)	2,351.83 (33.4%)	
2011–2014	4,877.69 (49.01%)	7,167.14 (50.94%)	3,562.59 (50.6%)	
**Primary site**				0.0220
Colon	5,986.40 (60.15%)	8,641.89 (61.42%)	4,285.85 (60.87%)	
Rectosigmoid	764.36 (7.68%)	1,160.58 (8.25%)	587.97 (8.35%)	
Rectum	3,201.45 (32.17%)	4,268.67 (30.34%)	2,167.52 (30.78%)	
**Stage**				0.0627
II	3,678.71 (36.96%)	4,809.89 (34.18%)	2,437.08 (34.61%)	
III	6,250.42 (62.8%)	9,230.41 (65.6%)	4,592.69 (65.22%)	
Unknown	23.08 (0.23%)	30.84 (0.22%)	11.57 (0.16%)	
**Grade**				0.0583
Well or moderately differentiated	8,702.29 (87.44%)	12,231.23 (86.92%)	6,116.67 (86.87%)	
Poorly differentiated	798.83 (8.03%)	1,232.69 (8.76%)	612.12 (8.69%)	
Unknown	451.09 (4.53%)	607.23 (4.32%)	312.55 (4.44%)	
**Primary treatment**				0.1472
OP alone	0 (0%)	0 (0%)	0 (0%)	
OP-CCRT	652.58 (6.56%)	925.74 (6.58%)	455.80 (6.47%)	
OP-CT	8,121.55 (81.61%)	11,466.29 (81.49%)	5,742.30 (81.55%)	
Neo-CCRT	612.75 (6.16%)	856.59 (6.09%)	433.28 (6.15%)	
Neo-CT	43.30 (0.44%)	63.41 (0.45%)	34.04 (0.48%)	
Unknown + missing	522.03 (5.25%)	759.11 (5.39%)	375.92 (5.34%)	
**Comorbidity**				
Hypertension	5,131.89 (51.57%)	7,078.44 (50.3%)	3,535.89 (50.22%)	0.0270
Dyslipidemia	2,538.28 (25.5%)	3,406.76 (24.21%)	1,734.15 (24.63%)	0.0299
Diabetes mellitus	2,637.37 (26.5%)	3,631.37 (25.81%)	1,821.48 (25.87%)	0.0158
Coronary artery disease	3,461.63 (34.78%)	4,884.72 (34.71%)	2,434.68 (34.58%)	0.0043
Chronic obstructive pulmonary disease	211.47 (2.12%)	264.23 (1.88%)	133.51 (1.9%)	0.0176
Peripheral arterial disease	603.16 (6.06%)	797.19 (5.67%)	413.71 (5.88%)	0.0168
Chronic kidney disease	1,910.97 (19.2%)	2,573.97 (18.29%)	1,290.95 (18.33%)	0.0233
Atrial fibrillation	355.08 (3.57%)	480.38 (3.41%)	218.21 (3.1%)	0.0261
Moderate or severe liver disease	17.50 (0.18%)	21.73 (0.15%)	12.32 (0.17%)	0.0053
**Postdiagnostic medication**				
Aspirin	1,670.38 (16.78%)	2,263.41 (16.09%)	1,150.93 (16.35%)	0.0189
Metformin	1,117.73 (11.23%)	1,547.76 (11%)	801.37 (11.38%)	0.0121
Statin	1,469.83 (14.77%)	2,038.47 (14.49%)	1,021.51 (14.51%)	0.0080

*ASMD, absolute standardized mean difference; EC1, civil servants: full-time or regularly paid personnel with a government or public affiliation; EC2, employees of privately owned institutions; EC3, self-employed individuals, other employees, and members of farmers' or fishermen's associations; EC4, veterans, members of low-income families, and substitute service draftees; CCRT, chemotherapy and radiotherapy; CT, chemotherapy; OP, operation; SIPTW, stabilized inverse probability of treatment weighting using propensity score; UFT, fluoropyrimidine*.

**Figure 3:**
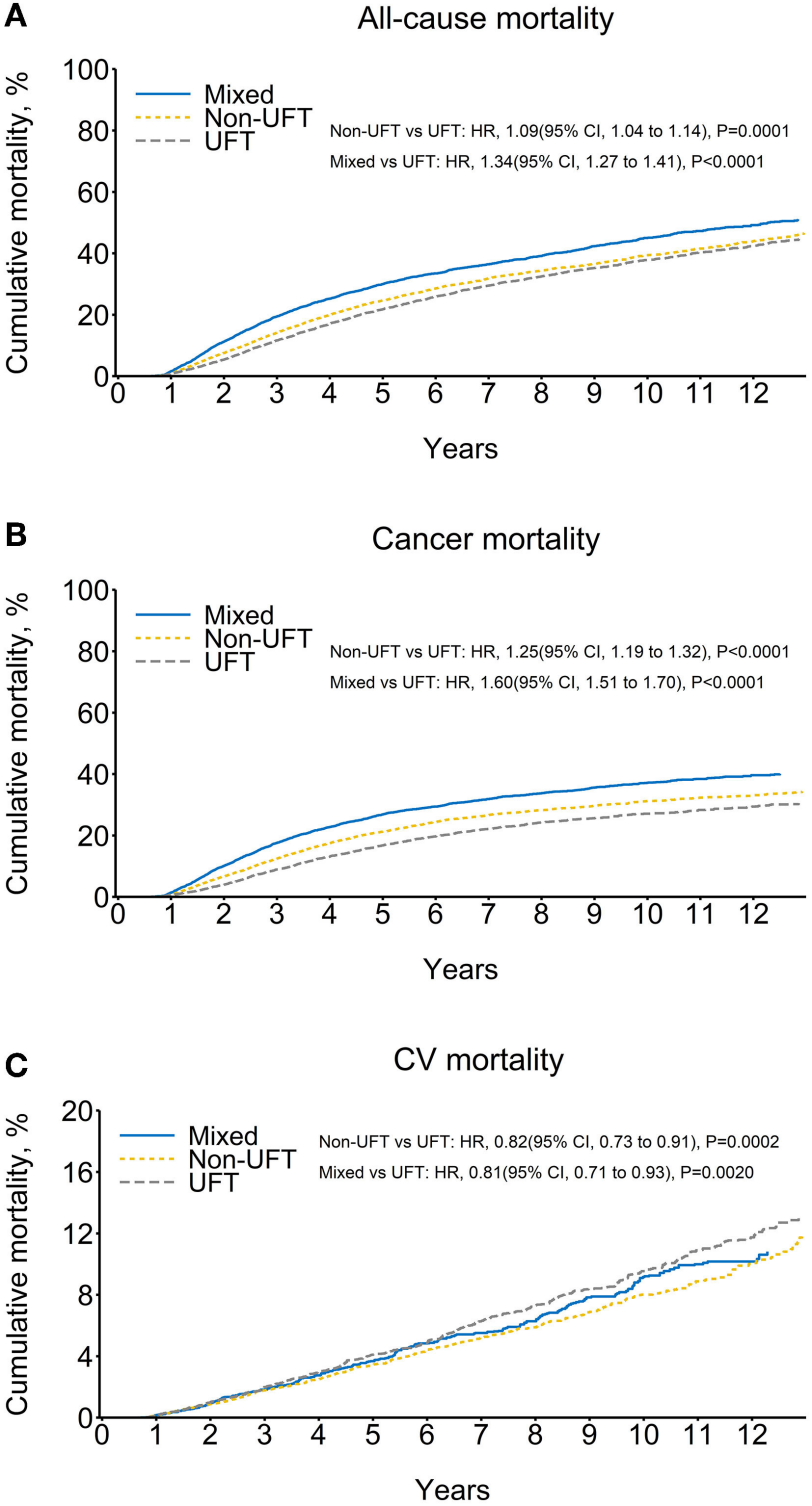
Cumulative mortality among patients with stage II–III colorectal cancer, after SIPTW (SIPTW, stabilized inverse probability of treatment weighting using propensity score; UFT, fluoropyrimidine).

Before SIPTW, there were 3,367, 4,672, and 2,693 all-cause deaths in the UFT, non-UFT, and mixed groups, respectively, equivalent to all-cause mortality of 5.00, 4.95, and 6.14 per 100 person-years. The mixed group had a significantly higher risk of all-cause mortality than the UFT group (HR = 1.24, 95% CI = 1.18–1.30). Cancer was still the leading cause of death, and the cancer death rates per 100 person-years were 3.12, 4.03, 5.11 for the UFT, non-UFT, and mixed groups, respectively. The non-UFT group (HR = 1.36, 95% CI = 1.29–1.43) and the mixed group (HR = 1.70, 95% CI = 1.61–1.81) had significantly more cancer deaths than the UFT group. CV events accounted for 23.5, 12.9, and 12.0% of all-cause deaths, and the CV death rates per 100 person-years were 1.17, 0.64, and 0.74 in the UFT, non-UFT, and mixed groups, respectively. The non-UFT (HR = 0.53, 95% CI = 0.47–0.58) and mixed groups (HR = 0.58, 95% CI = 0.51–0.66) had significantly lower CV death rates than the UFT group (Supplementary Figure 1). After SIPTW, the differences in all-cause mortality, cancer death rate, and CV death rates between the three CT groups were similar to those before SIPTW, except that a significantly higher all-cause mortality was observed in the non-UFT group than in the UFT group (HR = 1.09, 95% CI = 1.04–1.14) (Figure 3; Table 3).

**Table 3:** Mortality among patients with stage II–III colorectal cancer before and after SIPTW.

	**Before SIPTW**			**After SIPTW**			**Before SIPTW**		**After SIPTW**	
	**No. of** **event**	**Person-years**	**Incidence** **rate**	**No. of** **event**	**Person-years**	**Incidence** **rate**	**Hazard** **ratio**	***p*** **-value**	**Hazard** **ratio**	***p*** **-value**
**All-cause death**										
UFT	3,367	67,378.74	5.00 (4.83–5.17)	3,103.3	66,102.60	4.69 (4.53–4.86)	Reference		Reference	
Non-UFT	4,672	94,472.91	4.95 (4.80–5.09)	4,618.13	90,038.84	5.13 (4.98–5.28)	1.00 (0.96–1.04)	0.9505	1.09 (1.04–1.14)	0.0001
Mixed	2,693	43,884.61	6.14 (5.90–6.37)	2,672.06	42,630.69	6.27 (6.03–6.51)	1.24 (1.18–1.30)	<0.0001	1.34 (1.27–1.41)	<0.0001
**Cancer death**										
UFT	2,099	67,378.74	3.12 (2.98–3.25)	2,134.3	66,102.60	3.23 (3.09–3.37)	Reference		Reference	
Non–UFT	3,804	94,472.91	4.03 (3.90–4.15)	3,608.62	90,038.84	4.01 (3.88–4.14)	1.36 (1.29–1.43)	<0.0001	1.25 (1.19–1.32)	<0.0001
Mixed	2,243	43,884.61	5.11 (4.90–5.32)	2,191.25	42,630.69	5.14 (4.92–5.36)	1.70 (1.61–1.81)	<0.0001	1.60 (1.51–1.70)	<0.0001
**CV death**										
UFT	790	67,378.74	1.17 (1.09–1.25)	611.78	66,102.60	0.93 (0.85–1.00)	Reference		Reference	
Non-UFT	602	94,472.91	0.64 (0.59–0.69)	700.16	90,038.84	0.78 (0.72–0.84)	0.53 (0.47–0.58)	<0.0001	0.82 (0.73–0.91)	0.0002
Mixed	324	43,884.61	0.74 (0.66–0.82)	348.58	42,630.69	0.82 (0.73–0.90)	0.58 (0.51–0.66)	<0.0001	0.81 (0.71–0.93)	0.0020

*CV, cardiovascular; SIPTW, stabilized inverse probability of treatment weighting using propensity score; UFT, fluoropyrimidine; The hazard ratio was obtained using Cox's proportional hazard model and chemotherapy (CT) grouping was the only covariate included because the three CT groups were balanced after SIPTW. () represent then 95% confidence interval*.

**Figure 4:**
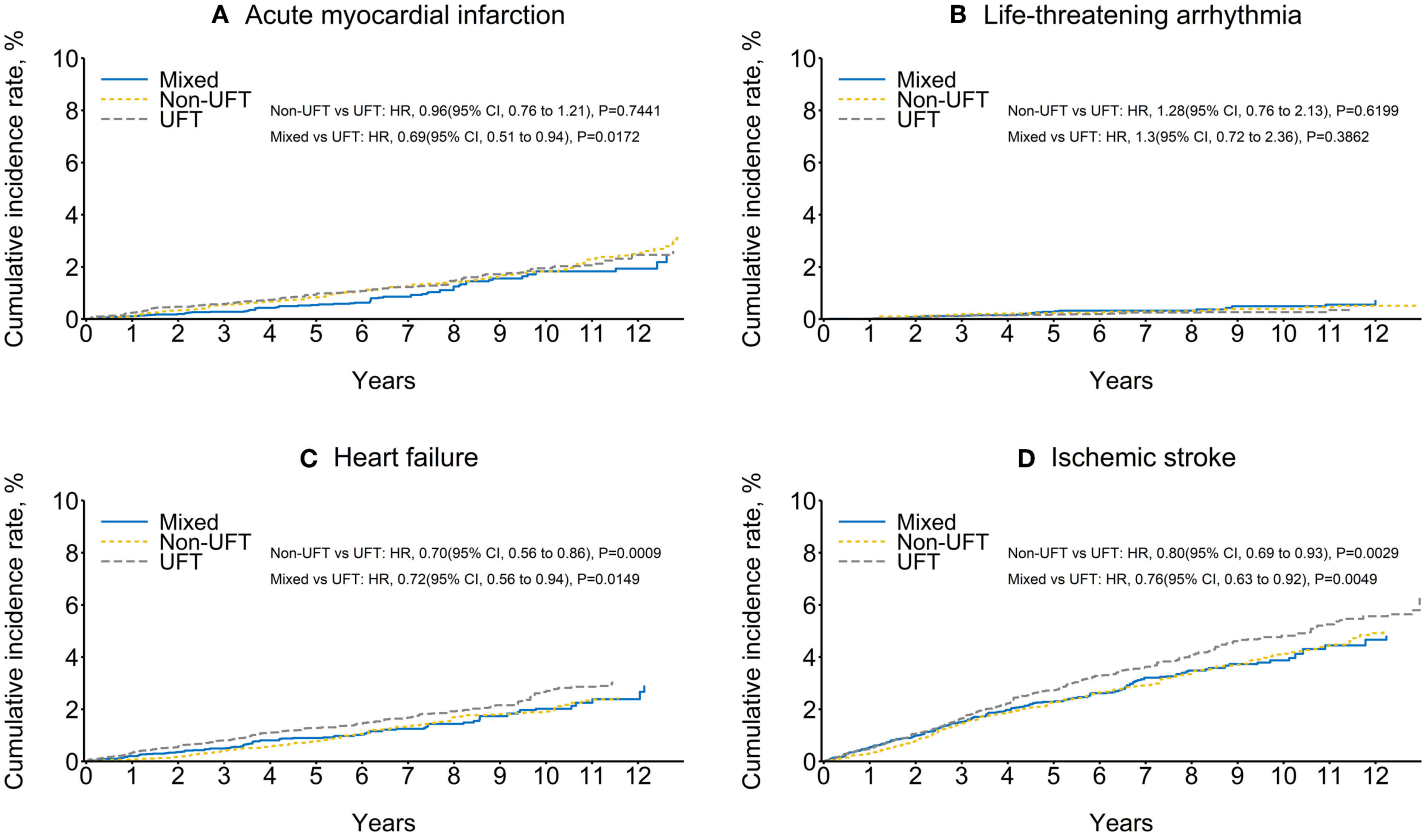
Cumulative rate of cardiovascular outcomes among patients with stage II–III colorectal cancer after SIPTW (SIPTW, stabilized inverse probability of treatment weighting using propensity score; UFT, fluoropyrimidine).

The ischemic stroke had the highest CV outcomes, followed by heart failure, AMI, and life-threatening arrhythmia. The non-UFT and mixed groups had significantly lower ischemic stroke and heart failure rates than those in the UFT group before and after SIPTW. There was no significant difference in life-threatening arrhythmias between the three CT groups before or after SIPTW. The non-UFT group and the mixed group had a significantly lower AMI rate than the UFT group before SIPTW (Supplementary Figure 2), but the difference was non-significant between the non-UFT and UFT groups after SIPTW (HR = 0.96, 95% CI = 0.76–1.21) (Figure 4; Table 4).

**Table 4:** Cardiovascular outcomes among patients with stage II–III colorectal cancer before and after SIPTW.

	**Before SIPTW**			**After SIPTW**			**Before SIPTW**		**After SIPTW**	
	**No. of event**	**Person years**	**Incidence rate**	**No. of event**	**Person years**	**Incidence rate**	**Sub-hazard ratio**	***p*** **-value**	**Sub-hazard ratio**	***p*** **-value**
**Acute myocardial infarction**										
UFT	155	67,006.37	0.23 (0.19–0.27)	126.42	65,769.99	0.19 (0.16–0.23)	Reference		Reference	
Non-UFT	159	94,001.24	0.17 (0.14–0.20)	170.72	89,569.80	0.19 (0.16–0.22)	0.71 (0.57–0.88)	0.0022	0.96 (0.76–1.21)	0.7441
Mixed	59	43,727.66	0.13 (0.10–0.17)	61.03	42,484.72	0.14 (0.11–0.18)	0.54 (0.40–0.73)	< .0001	0.69 (0.51–0.94)	0.0172
Life–threatening arrhythmia										
UFT	31	67,362.05	0.05 (0.03–0.06)	22.69	66,090.27	0.03 (0.02–0.05)	reference		reference	
Non–UFT	31	94,441.10	0.03 (0.02–0.04)	40.71	89,983.29	0.05 (0.03–0.06)	0.70 (0.42–1.14)	0.1532	1.28 (0.76–2.13)	0.3513
Mixed	21	43,869.56	0.05 (0.03–0.07)	20.71	42,618.10	0.05 (0.03–0.07)	0.97 (0.56–1.68)	0.9026	1.30 (0.72–2.36)	0.3862
**Heart failure**										
UFT	231	66,884.73	0.35 (0.30–0.39)	167.52	65,759.45	0.25 (0.22–0.29)	Reference		Reference	
Non–UFT	145	94,122.35	0.15 (0.13–0.18)	163.86	89,682.55	0.18 (0.15–0.21)	0.44 (0.35–0.54)	<0.0001	0.70 (0.56–0.86)	0.0009
Mixed	81	43,708.12	0.19 (0.14–0.23)	84.86	42,446.16	0.20 (0.16–0.24)	0.50 (0.39–0.64)	<0.0001	0.72 (0.56–0.94)	0.0149
**Ischemic stroke**										
UFT	418	65,992.73	0.63 (0.57–0.69)	336.67	64,953.99	0.52 (0.46–0.57)	Reference		Reference	
Non–UFT	330	93,334.74	0.35 (0.32–0.39)	380.88	88,775.08	0.43 (0.39–0.47)	0.55 (0.48–0.64)	<0.0001	0.80 (0.69–0.93)	0.0029
Mixed	164	43,223.62	0.38 (0.32–0.44)	182.89	41,914.30	0.44 (0.37–0.50)	0.56 (0.47–0.67)	<0.0001	0.77 (0.64–0.92)	0.0049

*SIPTW, stabilized inverse probability of treatment weighting using propensity score; UFT, fluoropyrimidine; The sub-hazard ratio was obtained using the cause-specific hazard models and chemotherapy (CT) grouping was the only covariate included because the three CT groups were balanced after SIPTW. () represent then 95% confidence interval*.

**Figure 5:**
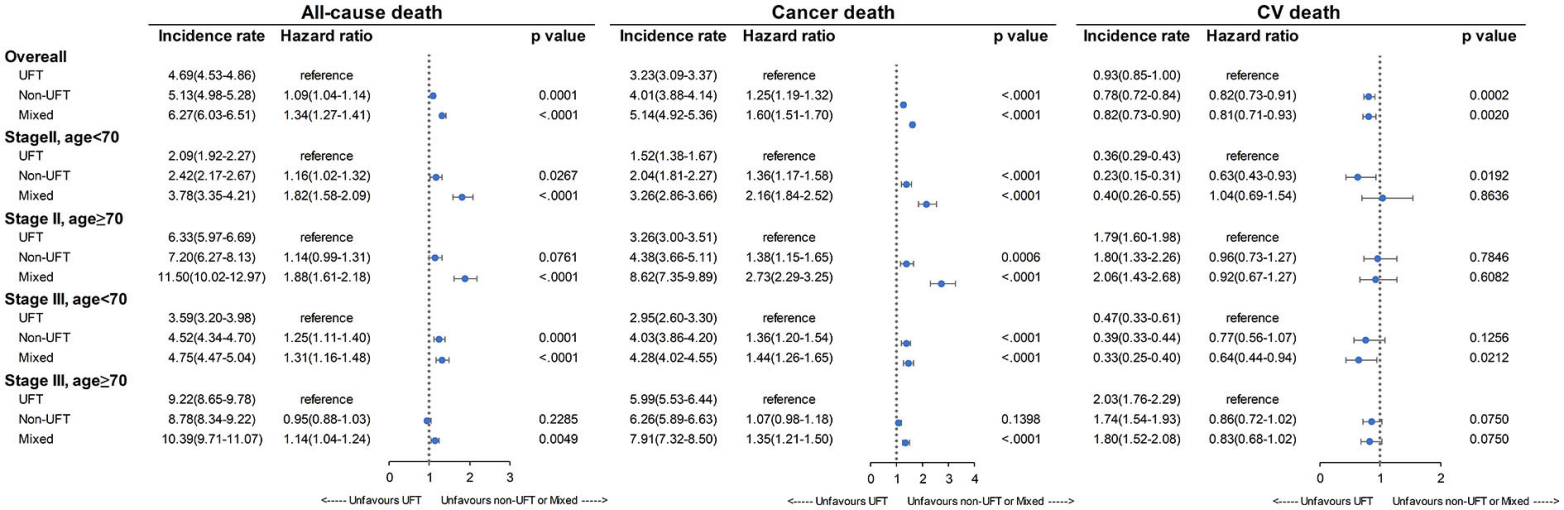
Mortality rate among patients with stage II–III colorectal cancer after SIPTW subgroup analysis (SIPTW, stabilized inverse probability of treatment weighting using propensity score; UFT, fluoropyrimidine).

Figure 5 presents the mortality results of subgroup analysis for stage II and age <70 years, stage II and age ≥70 years, stage III and age <70 years, and stage III and age ≥70 years. Again, the UFT group had significantly lower all-cause and cancer mortality rates than the other two CT groups, except for stage III and age ≥70 years and the non-UFT users in the group of stage II and age ≥70 years (*p* = 0.0761). In contrast, the UFT group had higher CV mortality than the non-UFT group and reached significance in the stage II and age <70 years subgroup.

**Figure 6:**
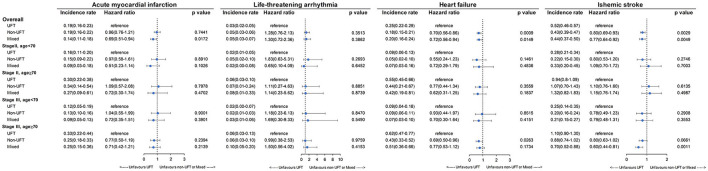
Incidence rate of cardiovascular outcomes among patients with stage II–III colorectal cancer after SIPTW subgroup analysis (SIPTW, stabilized inverse probability of treatment weighting using propensity score; UFT, fluoropyrimidine).

Figure 6 presents the CV outcomes of subgroup analysis for stage II and age <70 years, stage II and age ≥70 years, stage III and age <70 years, and stage III and age ≥70 years. There was no significant difference among the three CT groups for the four subgroup analyses for AMI, LTA, heart failure, and ischemic stroke, except for the following: the UFT group had a significantly higher heart failure rate than the non-UFT groups in those with stage III and age ≥70 years (*p* = 0.0263). However, for ischemic stroke, a marginally higher rate in the UFT group than in the non-UFT group was seen only in those with stage III disease and aged ≥70 years (*p* = 0.0661).

## Discussion

Although the survival benefit of adjuvant chemotherapy in patients with high-risk stage II or III CRC is generally accepted (21–23), cardiotoxicity from 5-FU derivatives may compromise the quality of life and overall survival. Therefore, this study explored the association between cardiotoxicity and fluoropyrimidine-based adjuvant chemotherapy in CRC patients. Mechanistically, two hypotheses of 5-FU-related cardiotoxicity included (1) dihydropyrimidine dehydrogenase (DPD) downstream metabolites and (2) 5-FU direct injury to endothelial cells (24).

First, oral fluoropyrimidines, including TS-1 and UFT, may affect the risk of cardiotoxicity by regulating DPD enzymes. DPD enzyme activity related to cardiotoxicity has been reported previously (25). The downstream metabolites are fluoroacetate and F-citrate, which have been related to potent cardiotoxicity in previous studies (26, 27). Second, according to our hypothesis, UFT, an adjuvant chemotherapeutic agent, inhibits DPD with uracil and may decrease CV events. However, our results did not support this hypothesis.

Second, 5-FU can directly damage endothelial cells, causing vasoconstriction and thromboembolism. Previous studies have reported that continuous intravenous injection (2–18%) is more likely to induce cardiotoxicity than a bolus regimen (1.6–3.0%) (28, 29). Another oral prodrug, capecitabine, is 5.9% in cardiotoxicity (30). Therefore, prodrug or continuous intravenous injection may prolong 5-FU toxic exposure, inducing more endothelial cell damage. UFT was a prodrug that increased 5-FU toxicity exposure time in our study. Our results revealed a continuing need for a mechanism of cardiotoxicity induced by metabolites in this pathway. Much more also needs to be known about the role of uracil and DPD in cardiotoxicity. Future work will hopefully clarify the mechanism of 5-FU-induced cardiotoxicity.

Several studies have examined the association between 5-FU-based adjuvant chemotherapy and cardiotoxicity. However, there are no retrospective studies on a large number of patients with CRC comparing different 5-FU-based adjuvant chemotherapy regimens. Our study took steps to compare different regimens in a nationwide cohort. UFT did not decrease the possibility of induced cardiotoxicity. Oral UFT, the most common and clinically prescribed drug in outpatients, may be associated with a higher likelihood of CV events. Further surveys of heart function should be considered before UFT use, especially in older adults and advanced-stage disease.

The first limitation of our study is selection bias. Oncologists may consider performance status and, therefore, prescribe different 5-FU derivatives. In addition, higher comorbidities were noted in the UFT group. Patients in poor condition may prefer oral UFT. Another limitation is that potential confounders were not recorded in the Taiwan's National Health Insurance Research Database. Individual demographic and lifestyle factors, including performance status, BMI, smoking, and exercise activity, may be associated with the development of CV events. Lastly, we did not have data of UFT or other FU dose for further analysis of dose-event relationship.

## Conclusion

In conclusion, UFT use was associated with a higher CV events and deaths rate than adjuvant chemotherapy-induced cardiotoxicity. Specifically, the subgroup analysis revealed higher CV events in the UFT group, older patients, and stage III patients. The present study provides the first comparative analysis of cardiotoxicity between UFT and other 5-FU derivatives. Our results suggest that caregivers should be alert to UFT use in older patients with multiple comorbidities. Further research is needed to develop a risk prediction tool to stratify patients and guide the choice of 5-FU regimens accordingly.

## Data availability statement

We used the Taiwan Cancer Registry, National Health Insurance Research Database Taiwan, Taiwan Death Registry, which are only available in the Health and Welfare Data Science Center, Taiwan. We cannot make our research data available, accessible, discoverable, and usable.

## Ethics statement

The study was approved by the Institutional Review Board of the Chang Gung Medical Foundation (201901844B0). Informed consent was waived because all personal identification information was encrypted.

## Author contributions

W-KH, W-PH, and L-CS contributed to the conceptualization and drafted the manuscript. W-KH, W-PH, H-CH, and L-CS designed this study. S-HC and L-CS performed data curation. S-HC, W-KH, and L-CS performed formal analyses. W-KH, H-CH, W-CC, and S-HC are involved in the resources and software development. D-YC, W-CC, P-HC, T-SY, and J-SC contributed to project administration. L-CS was responsible for the funding acquisition. W-KH, W-PH, H-CH, S-HC, D-YC, W-CC, P-HC, J-SC, T-SY, and L-CS contributed to the review and editing of this manuscript. All authors contributed to the article and approved the submitted version.

## Funding

This study was funded by grants NMRPD1K0771 and CMRPD1K0022 from the Chang Gung Medical Foundation and grant MOST 109-2314-B-182-035 from the Ministry of Science and Technology, Taiwan.

## Conflict of interest

The authors declare that the research was conducted in the absence of any commercial or financial relationships that could be construed as a potential conflict of interest.

## Publisher's note

All claims expressed in this article are solely those of the authors and do not necessarily represent those of their affiliated organizations, or those of the publisher, the editors and the reviewers. Any product that may be evaluated in this article, or claim that may be made by its manufacturer, is not guaranteed or endorsed by the publisher.
