# Supplementary material for: Risk of cardiovascular disease among different fluoropyrimidine-based chemotherapy regimens as adjuvant treatment for resected colorectal cancer
Source: Front Cardiovasc Med. 2022 Aug 3;9:880956. doi: 10.3389/fcvm.2022.880956 (PMC9381971; doi:10.3389/fcvm.2022.880956)
Supplement: Supplementary file 1 [file Table_1.docx]

Supplemental Table 1. The ICD-9 and ICD-10 for the four study outcomes

| Study outcomes | ICD-9 | ICD-10 |
| --- | --- | --- |
| acute myocardial infarction (AMI) | 410.X | I2109, I2119, I2111, I2129, I214, I213, |
| life-threatening arrhythmia (LTA) | 427.1, 427.4, 427.5 | I472, I4901, I4902, I469 |
| congestive heart failure (CHF) | 428.X | I509, I501, I5020, I5021, I5022, I5023, I5030, I5031, I5032, I5033, I5040, I5041, I5042, I5043, I509 |
| ischemic stroke (IS) | 433, 434, 435 | I651, I6322, I6529, I63139, I63239, I6509, I63019, I63119, I63219, I658, I6359, I658, I6359, I659, I6320, I6609, I6619, I6629, I6330, I6609, I6619, I6629, I669, I6340, I669, I6350, G450, G451, G458, G459, I67848 |
